# Minimally Symptomatic Atrial Fibrillation Patients Derive Significant Symptom Relief Following Rate Control or Rhythm Control Therapy

**DOI:** 10.14740/jocmr2209w

**Published:** 2015-07-24

**Authors:** David Ryan King, Neil D. Mehta, Anil K. Gehi, Irion Pursell, Paul Mounsey, Prabhat Kumar, Ayo Bamimore, Eugene H. Chung

**Affiliations:** a UNC Center for Heart and Vascular Care, Department of Medicine, Cardiac Electrophysiology, University of North Carolina at Chapel Hill, NC, USA

**Keywords:** Antiarrhythmic drugs, Atrial fibrillation, Electrophysiology, Heart

## Abstract

**Background:**

It can be challenging to convince asymptomatic to minimally symptomatic patients to pursue treatment of their atrial fibrillation (AF). We hypothesized that once in sinus rhythm, asymptomatic to minimally symptomatic patients would realize they were compensating for moderate symptoms, and that we could quantify this via the Canadian Cardiovascular Society Severity of AF (CCS-SAF) score.

**Methods:**

All patients in our study come from the Symptom Mitigation in Atrial Fibrillation (SMART) study. Upon enrollment all patients were assigned a CCS-SAF score. Patients receiving a CCS-SAF score of 0 or 1 that elected to pursue intervention were contacted by phone and asked about their symptoms post-intervention as compared to pre-intervention. Paired *t*-test was used for analysis.

**Results:**

Out of 800 patients in the SMART study to date, 48 patients have qualified for our phone survey and presented for follow-up in our clinic. In our cohort, the revised pre-intervention CCS-SAF score was 1.69 ± 1.36 and the post-intervention CCS-SAF score was 0.52 ± 0.80. Thirty-seven patients reported symptom improvement; those who improved were on average 72.4% improved from baseline.

**Conclusions:**

We conclude asymptomatic to minimally symptomatic AF patients benefit from therapy and should be offered intervention despite lack of symptoms.

## Introduction

Atrial fibrillation (AF) is the most common arrhythmia in the world affecting 1-2% of the general population [1]. AF incidence is expected to more than double, from 1.2 million cases in 2010 to 2.6 million cases in 2030, consequently AF prevalence is projected to increase from 5.2 million in 2010 to 12.1 million cases in 2030 [2]. It is known that AF increases the risk of stroke; therefore, the first priority is stroke prophylaxis via oral anticoagulation if the patient is a suitable candidate [3]. Then, consideration should be made whether intervention via rate or rhythm control therapy should be attempted or whether referral to an electrophysiology (EP) clinic is necessary. It has been shown that managing AF patients in specialty clinics reduces the incidence of AF-related hospitalizations and stroke [4].

Of particular concern is the newly diagnosed asymptomatic AF patient. About 33% of patients are unaware of having AF and thus may have delayed diagnosis and treatment [5]. This is of particular importance because increased rates of stroke and death have been associated with patients in AF [6]. Moreover, patients who deny symptoms may have grown tolerant of their condition, or do not realize the extent of their symptoms. Hence, it can be challenging to advise a new, asymptomatic patient to pursue treatment of their AF.

We hypothesized that asymptomatic to minimally symptomatic patients would realize the true extent of their symptoms and the amount of symptom compensation once they converted to sinus rhythm. We quantified this using the Canadian Cardiovascular Society Severity of AF (CCS-SAF) score and follow-up phone calls with these patients.

## Methods

The SMART study is a single-center prospective cohort study of patients with AF, measuring AF symptoms and health outcomes. Details of the SMART study have been previously described [7]. In brief, participants were enrolled through Outpatient Electrophysiology Clinics at the University of North Carolina Chapel Hill when referred for management of AF. Participants were excluded if they were planning to move from the local area within 3 years of enrollment or were less than 18 years of age. The appropriate institutional review board approved the study and all participants provided written informed consent.

Upon enrollment, participants completed a baseline questionnaire of general demographic information and measures of physical and psychological health and were assigned a baseline CCS-SAF score by the attending physician. Patients receiving a CCS-SAF score of 0 - 1 also completed a follow-up phone call questionnaire at least 6 months after initiation of intervention, asking: 1) Since your treatment for atrial fibrillation, have your symptoms improved? If so, can you estimate the percentage improvement on a scale from 0% to 100%? 2) On a scale of 0 - 4, 0 representing no symptoms and 4 the worst possible symptoms, how would you rate your symptom level today (patient-assigned SAF score)? 3) At your initial visit to the EP clinic (pre-treatment) your provider assigned you a score of _, on a scale of 0 - 4. In hindsight, knowing your symptom level today, was that number accurate? If no, would you want to revise it?

Baseline demographics and follow-up data were securely archived using REDCap, an online database used for data collection. Paired *t*-test was used for statistical analysis.

## Results

Of the 800 patients enrolled in the SMART study to date, 82 patients have qualified as asymptomatic or minimally symptomatic; 48 patients have returned for follow-up in our EP clinic and have complete data available. The 48 patients with complete data available formed our final study group. Baseline demographics for the patients included in the discussion, as well as patients excluded from the study, are provided in Table 1 and were statistically no different. Of the included 48 patients, 18 patients (37.5%) received rate control therapy and the remaining 30 patients (62.5%) received rhythm control therapy by way of antiarrhythmic medications, or a combination of antiarrhythmic medications plus catheter ablation. The patients were placed on rate control or rhythm control therapy based on what their provider believed to best confer to the patients’ needs, independent of our study.

**Table 1:** Baseline Demographics for Patients Included in Discussion and Patients Excluded From Discussion

	Patients included (n = 48)	Patients excluded (n = 34)	
Age (years)	71.1 ± 8.80	68.2 ± 16.2	NS
White	93.75% (45)	85% (29)	NS
Male	65% (31)	68% (23)	NS
BMI	30.48 ± 6.28	32.13 ± 9.19	NS
HTN	41.67% (20)	44% (15)	NS
CHF	6.25% (3)	12% (4)	NS
Beta blocker	60.4% (29)	68% (23)	NS
CCB	18.8% (9)	24% (8)	NS
ACE/ARB	29.2% (14)	41% (14)	NS
Statin	35.4% (17)	47% (16)	NS
Persistent AF	29.2% (14)	35% (12)	NS
Baseline AF burden	44.8±46.95%*	54.73±48.91%**	NS
CHADS2 score	1.77 ± 1.36	1.41 ± 1.10	NS
CHADS-VASc score	2.85 ± 1.70	2.44 ± 1.58	NS

Comparison between the two groups yielded non-significant (NS) difference in each parameter. *Baseline AF burden available in only 42 patients included in discussion; **Baseline AF burden available in only 28 patients excluded from discussion.

Patients were contacted by phone 499 ± 250 days after initiation of treatment and asked to assign a retrospective pre-intervention CCS-SAF score and a post-intervention CCS-SAF score. These scores were 1.69 ± 1.36 and 0.52 ± 0.80 respectively (P < 0.0001, not shown in figure). Symptoms improved in 37 of the 48 (77.1%) patients surveyed; when asked to quantify their improvement from 0% improved to 100% improved, those who improved were on average 72.4% improved from baseline.

**Figure 1:**
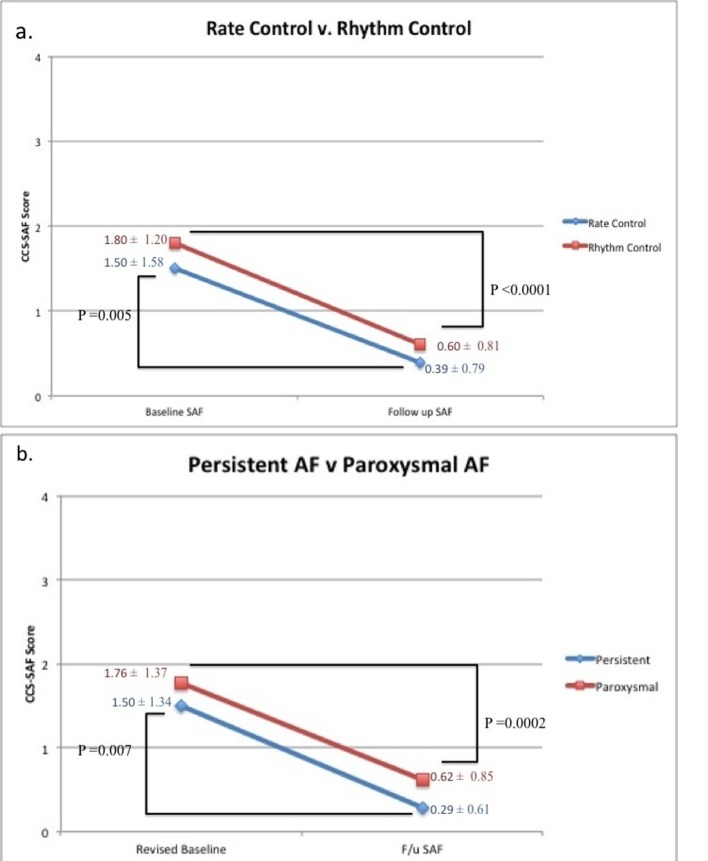
Summarization of treatment effect on CCS-SAF score in subpopulations in our low SAF cohort. (a) Our subpopulations of rhythm control (n = 30) and rate control (n=18). (b) Our subpopulations of persistent AF (n = 14) and paroxysmal AF (n = 34).

Furthermore, we examined CCS-SAF score improvement in patients who received rate control (18 patients), versus those who received rhythm control (30 patients). In both populations significant CCS-SAF score improvement was observed (P = 0.005 and P < 0.0001 respectively) (Fig. 1a). Also, we examined CCS-SAF improvement in patients suffering from paroxysmal AF and persistent AF at baseline. Again, in both populations significant CCS-SAF score improvement was observed (P = 0.0002 and P = 0.007 respectively) (Fig. 1b).

## Discussion

In our cohort, minimally symptomatic patients benefitted from rhythm control therapy once converted to sinus rhythm (P < 0.0001; Fig. 1a). Unexpectedly, we also found patients on rate control therapy derived significant symptom improvement as well (P = 0.005; Fig. 1a). Since we first presented our data, asymptomatic longstanding persistent AF patients have been found to experience symptom relief following catheter ablation [8, 9]. Our results demonstrate that in addition to persistent AF patients, paroxysmal AF patients also derive significant symptom reduction from intervention (P = 0.0002; Fig. 1b). Our results suggest asymptomatic to minimally symptomatic AF patients compensate for their symptoms at baseline and achieve symptom mitigation following intervention.

Patients living with untreated AF are at risk for embolic events. Paroxysmal AF, if transient, infrequent, and sometimes asymptomatic, may be undetected on continuous telemetry and 24 or 48-h Holter monitors. A prospective study of 2,580 subjects, age ≥ 65 years, with hypertension and no history of AF who had recent implantation of a pacemaker or defibrillator (ASSERT) links subclinical AF and cryptogenic stroke [10]. In another study among subjects with at least 3 months of continuous monitoring who experienced ischemic stroke or systemic embolism (n = 51), subclinical AF was detected in 26 (51%) subjects [11]. These results suggest that there is no temporal relationship between asymptomatic or undetected AF and stroke; however, it is shown that subclinical AF is associated with an increased risk of embolic events.

Recent studies support enhanced surveillance in patients who experience cryptogenic stroke or TIA for several weeks to rule out subclinical AF. In the CRYSTAL AF trial, 441 patients with cryptogenic stroke and no evidence of AF during 24 h of continuous ECG monitoring were randomly assigned to prolonged monitoring using an implantable loop recorder (monitored group) or a conventionally monitored control group [12]. At 6 months, AF detection was significantly higher in the monitored group (8.9%) and 1.4% in the control group (hazard ratio 6.4, 95% CI: 1.9 - 21.7). Results were similar in the EMBRACE trial [13]. These studies confirm that prolonged cardiac event monitoring can significantly increase the detection of idiopathic AF in patients who experience TIA or acute ischemic stroke.

In hopes of reducing the risk of embolic events and aiding patients with symptom mitigation, we would thus argue that asymptomatic to minimally symptomatic patients be urged to consider intervention despite appearing asymptomatic.

### Limitations

There were several limitations to the study. First, the patients were not randomized or subjected to uniform treatment. Second, a placebo effect must be considered, in AF, patients’ anxiety and depression can exacerbate the symptoms and treatment of any sort may lessen the anxiety a patient has in regard to their condition [14]. Lastly, the revised baseline SAF being assessed in retrospect makes it subject to recall bias.

### Conclusions

In the vast majority of our patients with asymptomatic to minimally symptomatic AF, the true extent of symptom compensation was realized following intervention. Appearing asymptomatic should not deter urging the patient to attempt rhythm control or rate control therapy for AF management. Treatment should be considered in all AF patients, including those presenting asymptomatic and minimally symptomatic.

## References

[1] Camm AJ, Lip GY, De Caterina R, Savelieva I, Atar D, Hohnloser SH, Hindricks G (2012). 2012 focused update of the ESC Guidelines for the management of atrial fibrillation: an update of the 2010 ESC Guidelines for the management of atrial fibrillation. Developed with the special contribution of the European Heart Rhythm Association. Eur Heart J.

[2] Colilla S, Crow A, Petkun W, Singer DE, Simon T, Liu X (2013). Estimates of current and future incidence and prevalence of atrial fibrillation in the U.S. adult population. Am J Cardiol.

[3] Olsson SB, Halperin JL (2005). Prevention of stroke in patients with atrial fibrillation. Semin Vasc Med.

[4] Tran HN, Tafreshi J, Hernandez EA, Pai SM, Torres VI, Pai RG (2013). A multidisciplinary atrial fibrillation clinic. Curr Cardiol Rev.

[5] Lin HJ, Wolf PA, Benjamin EJ, Belanger AJ, D'Agostino RB (1995). Newly diagnosed atrial fibrillation and acute stroke. The Framingham Study. Stroke.

[6] Hunter RJ, McCready J, Diab I, Page SP, Finlay M, Richmond L, French A (2012). Maintenance of sinus rhythm with an ablation strategy in patients with atrial fibrillation is associated with a lower risk of stroke and death. Heart.

[7] Gehi AK, Sears S, Goli N, Walker TJ, Chung E, Schwartz J, Wood KA (2012). Psychopathology and symptoms of atrial fibrillation: implications for therapy. J Cardiovasc Electrophysiol.

[8] Mohanty S, Santangeli P, Mohanty P, Di Biase L, Holcomb S, Trivedi C, Bai R (2014). Catheter ablation of asymptomatic longstanding persistent atrial fibrillation: impact on quality of life, exercise performance, arrhythmia perception, and arrhythmia-free survival. J Cardiovasc Electrophysiol.

[9] Chung EH, Pursell I, King DR Minimally Symptomatic Patients with Atrial Fibrillation are Actually Moderately Symptomatic and have Significant Symptom Reduction with Rhythm Control. Poster presentation, PO05-68, Heart Rhythm Scientific Sessions 2013.

[10] Healey JS, Connolly SJ, Gold MR, Israel CW, Van Gelder IC, Capucci A, Lau CP (2012). Subclinical atrial fibrillation and the risk of stroke. N Engl J Med.

[11] Brambatti M, Connolly SJ, Gold MR, Morillo CA, Capucci A, Muto C, Lau CP (2014). Temporal relationship between subclinical atrial fibrillation and embolic events. Circulation.

[12] Sanna T, Diener HC, Passman RS, Di Lazzaro V, Bernstein RA, Morillo CA, Rymer MM (2014). Cryptogenic stroke and underlying atrial fibrillation. N Engl J Med.

[13] Gladstone DJ, Spring M, Dorian P, Panzov V, Thorpe KE, Hall J, Vaid H (2014). Atrial fibrillation in patients with cryptogenic stroke. N Engl J Med.

[14] Lane DA, Langman CM, Lip GY, Nouwen A (2009). Illness perceptions, affective response, and health-related quality of life in patients with atrial fibrillation. J Psychosom Res.

